# The significance of polymorphisms in genes encoding Il-1β, Il-6, TNFα, and Il-1RN in the pathogenesis of intraventricular hemorrhage in preterm infants

**DOI:** 10.1007/s00381-017-3458-2

**Published:** 2017-06-29

**Authors:** Dawid Szpecht, Janusz Gadzinowski, Agnieszka Seremak-Mrozikiewicz, Grażyna Kurzawińska, Krzysztof Drews, Marta Szymankiewicz

**Affiliations:** 1 Chair and Department of Neonatology, Poznan University of Medical Sciences, Poznań, Poland; 2 Department of Perinatology and Women’s Diseases, Poznan University of Medical Sciences, Poznań, Poland; 3 Department of Pharmacology and Phytochemistry, Institute of Natural Fibers and Plants, Poznań, Poland

**Keywords:** Intraventricular hemorrhage, Gene polymorphism, Preterm neonates

## Abstract

**Introduction:**

Intraventricular hemorrhage (IVH) is a significant morbidity seen in very low birth weight infants. Genes related to inflammation may be risk factors for IVH.

**Material and methods:**

We examined five polymorphisms for an association with IVH in 100 preterm infants born from singleton pregnancy, before 32 + 0 weeks of gestation, exposed to antenatal steroid therapy, and without congenital abnormalities. These polymorphisms include interleukin-1β *3953* *C>T*, interleukin-6 −*174G>C* and −*596G>A*, tumor necrosis factor −*308* *G>A*, and 86 bp variable number tandem repeat polymorphism of interleukin-1 receptor antagonist (Il -1RN*﻿ 86 bp VNTR*).

**Results:**

In our study population, 45 (45%) infants developed IVH, including 15 (33.33%) with stage 1, 19 (42.22%) with stage 2, 8 (17.77%) with stage 3, and 3 (6.66%) with stage 4. In contrast to the previously published data, the prevalence of IVH did not vary between infants with different IL-6 and TNFα alleles and genotypes. Our novel investigations in Il-1 +*3953* *C>T* and Il-1RN* 86 bp VNTR* polymorphism did not show any significant link between those alleles or genotypes and IVH.

**Conclusions:**

IVH is a significant problem for preterm infants. In addition to little progress in preventing IVH in preterm babies, substantial research that are focused on understanding the etiology, mechanism and risk factors for IVH are imperative. In the era of personalized medicine, identification of genetic risk factors creates opportunities to generate preventative strategies. Further studies should be performed to confirm the role of genetic factors in etiology and pathogenesis of IVH.

**Electronic supplementary material:**

The online version of this article (doi:10.1007/s00381-017-3458-2) contains supplementary material, which is available to authorized users.

## Introduction

Intraventricular hemorrhage (IVH) is a condition which mainly affects infants born before 32 weeks of pregnancy. Approximately 90% of IVH cases occur within the first 3 days of the newborn’s life, and in 20–40% of cases, the spread of the initial bleeding becomes more extensive. IVH ranges in severity from grade 1 to the most severe grade 4. So far, the following risk factors for IVH have been described in literature: birth before 32 weeks of gestation, birth weight less than 1500 g, absence of prophylactic prenatal steroid therapy in women at risk of premature delivery, early clamping of the umbilical cord (up to 30 s after birth), symptoms of intrauterine infection in the mother and the newborn, and labor and delivery complicated by bleeding, perinatal hypoxia, respiratory distress syndrome (RDS), or patent ductus arteriosus (PDA) [1–3].

Contemporarily, it is thought that genetic factors may play a significant role in the development of IVH, but their connection with IVH in newborns has yet to be proven by any clinical studies. Undoubtedly, factors involved in the inflammatory processes such as interleukin-1β (Il-1β), interleukin 6 (Il-6), tumor necrosis factor alpha (TNFα), and interleukin-1 receptor antagonist (Il-1RN) play a role in the pathogenesis of IVH. IVH is multifactorial and complex complication of prematurity and primarily is attributed to the intrinsic fragility of the germinal matrix vasculature and the disturbance in the cerebral blood flow. Inflammation may directly or indirectly increase either the fluctuation in cerebral blood flow or the fragility of the germinal matrix microvasculature [3]. Neonatal infections are an important cause of morbidity and mortality and are associated with an increased risk of long-term neurological complications [4]. Genetic determination of the individual inflammatory response may influence the susceptibility to poor outcome after systemic infection or severe tissue damage [5]. Better understanding of the genetic factors that contribute to IVH and constitute a major role in its pathogenesis would be of great benefit in perinatal care to prevent brain injury and abnormal development.

The aim of this study was to evaluate the possible relationship between five polymorphisms in genes encoding Il-1β, Il-6, TNFα, and Il-1RN and the occurrence of IVH in a population of preterm newborns.

## Objectives

Between June 1, 2014, and August 15, 2016, 428 premature infants were born from 24 + 0 to 32 + 0 weeks of gestation and admitted to the neonatal intensive care unit at the Poznan University of Medical Sciences. Considering exclusion criteria, 100 (23.4%) out of the 432 infants were included in the study. In order to guarantee a homogenous ethnic background, all subjects were of Caucasian origin. The study did not include neonates born before 24 + 0 and after 32 + 0 weeks of pregnancy; multiple pregnancy neonates; pregnancies complicated by death of one of the fetuses; chromosomal abnormalities; toxoplasmosis, other, rubella, cytomegalovirus, herpes (TORCH) infections; inborn errors of metabolism; or infants without antenatal steroid therapy (AST).

## Materials and methods

### Clinical features

Studied perinatal factors that may interfere with the development of IVH include gender, gestational age (GA; weeks), birth weight (BW, grams), and small for gestational age (SGA, birth weight under the 3rd percentile). The type of delivery (vaginal birth vs. cesarean section), birth asphyxia (defined as Apgar score less than 6 at 10 min and pH <7.0 or blood base excess (BE) <−12 mmol/l in cord blood), and intrauterine infection (defined as positive culture in sterile blood originally accompanied by clinical symptoms or pneumonia developed in 48 h after the birth) could deter IVH development.

### Diagnosis

IVH was diagnosed with the use of a cranial ultrasound (10 MHz transducer, ProSound α7 Premier, Aloka). Routine cranial ultrasound examinations were performed on the 1st, 3rd, and 7th days after birth, in accordance with local standards and recommendations in infants born below 32 weeks gestation. Parents have consented to the cranial ultrasound. The maximal degree of IVH was confirmed by cranial ultrasound on the 7th day of life. The classification of intraventricular bleeding was based on the Papille IVH classification [6].

### Studied polymorphisms

We studied five single-nucleotide polymorphisms: Il-1β +*3953* *C>T*, Il-6 −*174G>C* and −*596G>A*, TNFα −*308G>A*, and 86 bp variable number tandem repeat polymorphism of interleukin-1 receptor antagonist (Il-1RN* VNTR 86 bp*).

A blood sample (0.5 ml) was taken directly post delivery and banked. Genomic DNA was extracted from blood leukocytes using QIAamp DNA Blood Mini Kit (Qiagen Inc, Germany). Genotyping was performed using polymerase chain reaction (PCR) procedures. The description of those studied polymorphisms in Il-1, Il-6, Il-1RN VNTR, and TNFα genes is shown in Table 1.

**Table 1:** Description of the five studied polymorphisms in Il-1, Il-6, Il-1RN, and TNFα genes

Gene symbol	Polymorphism	Sequence of primers	Restriction enzyme	Products
IL-1β (rs1143634)	+*3953* *C>T*	F 5′-gTTgTC ATC Aga CTT TgA CC-3′ R 5′-TTC AgT TCA TAT ggA CCA gA-3′	TaqI (Thermo Scientific)	*CC* 137,114 bp, *CT* 251,137,114 bp *TT* 251 bp
IL-1RN (rs2234663)	*86 bp VNTR*	F 5′-CTC AgC AAC ACT CCT AT-3′ R 5′-TCC Tgg TCT gCAggT AA-3′		IL1RN*0 154 bp IL1RN*1 410 bp IL1RN*2 240 bp IL1RN*3 500 bp IL1RN*4 325 bp IL1RN*5 595 bp
IL-6 (rs1800795)	−*174* *G>C*	F 5′-ACA TgC CAA gTgCTgAgT CA-3′ R 5′-AAT CTT TgTTggAgggTg Ag-3′	LweI (Thermo Scientific)	*GG* 114,100 bp *GC* 214,114,100 bp *CC* 214 bp
IL-6 (rs1800797)	−*596* *G>A*	F 5′-ggAgTC ACA CAC TCC ACC Tg-3′ R 5′-AAgCAg AAC CAC TCT TCC TTT ACT T-3′	BseGI (BtsCI) (Thermo Scientific)	*GG* 420 bp *GA* 420,354,66 bp *AA* 354, 66 bp
TNF-α (rs1800629)	−*308* *G>A*	5′-AAA TggAgg CAA Tag gTTTTgAggggCTTg-3′ 5′-TAC CCC TCA CAC TCC CCA TCC TCCCTg ATC-3′	FaqI (BsmFI) (Thermo Scientific)	GG 86,45 bp *GA* 131,86,45 bp *AA* 131 bp

Informed parental consent was obtained from each participating infant. The study followed the tenets of the Declaration of Helsinki and was approved by the Bioethics Committee of Poznan University of Medical Sciences (66/14 and 799/16). Furthermore, all methods and examinations were performed in accordance with the relevant ethical guidelines and regulations.

**Table 2:** Demographic and clinical characteristic of enrolled infants

	Group without IVH *N* = 55 (%)	Group with IVH grade 1-4 *N* = 45 (%)	*p* value	Group without IVH and with IVH grade 1 and 2 *N* = 89 (%)	Group with IVH grade 3–4 *N* = 11 (%)	*p* value
Gender						
Male	29 (52.73)	25 (55.56)	0.778^a^	47 (52.81)	7 (63.64)	0.719^b^
Female	26 (47.27)	20 (44.44)		42 (47.19)	4 (36.36)	
Gestational age (weeks)						
24 + 0–28 + 6	19 (34.55)	35 (77.78)	0.00002^s^	44 (49.44)	10 (90.91)	0.022^b^
29 + 0–32 + 0	36 (65.45)	10 (22.22)		45 (50.56)	1 (9.09)	
Birth weight (g)						
<750	3 (5.45)	11 (24.44)	0.0003^a^	12 (13.48)	2 (18.18)	0.057^a^
750–1000	10 (18.18)	17 (37.78)		21 (23.60)	6 (54.55)	
>1000	42 (76.37)	17 (37.78)		56 (62.92)	3 (27.27)	
Apgar score (median and range)						
1st minute	6 (1–10)	4 (1–10)	0.0001^c^	6 (1–10)	3 (1–7)	0.054^c^
5th minute	8 (5–10)	7 (1–9)	0.0005^c^	7 (2–10)	7 (1–8)	0.009^c^
Mode of delivery						
Vaginal	17 (29.09)	24 (53.33)	0.039^d^	35 (39.30)	6 (54.55)	0.520^d^
Cesarean section	38 (69.09)	21 (46.67)		54 (60.70)	5 (45.45)	
Asphyxia (pH >7.0 or BE <−12)						
Yes	0 (0.00)	3 (6.98)	0.084^e^	2 (2.30)	1 (10.00)	0.713^b^
No	54 (100.00)	40 (93.02)		85 (97.7)	9 (90.00)	
Intrauterine infection						
Yes	22 (40.00)	33 (73.33)	0.0009^a^	46 (51.69)	9 (81.82)	0.116^b^
No	33 (60.00)	12 (26.67)		43 (48.31)	2 (18.18)	
Deaths	2 (3.63)	8 (17.77)	0.038^b^	5 (5.6)	5 (45.45)	0.0004^b^

^a^Chi-square test

^b^Chi-square test with Yates’ correction

^c^Mann–Whitney *U* test

^d^Fisher–Freeman–Halton test

^e^Fisher’s exact test

### Statistical analysis

The results are presented as percentage for categorical variables, or median (range) for non-normally distributed continuous variables as tested by the Shapiro–Wilk test. A *p* value of less than 0.05 was considered significant. The Fisher exact probability test, the chi-squared test, the Fisher–Freeman–Halton test, and the chi-squared test with Yates correction were used to evaluate the association between IVH and categorical variables. Differences in non-normally distributed continuous variables were compared by the Mann–Whitney *U* test. The expected genotype frequencies were calculated from allele frequencies with the Hardy-Weinberg equilibrium. Statistical analysis was performed using CytelStudio version 10.0, created on January 16, 2013 (CytelStudio Software Corporation, Cambridge, MA, USA), and Statistica version 10, 2011 (StatSoft, Inc., Tulsa, OK, USA).

## Results

We found that 45 (45%) infants developed IVH in our study population, including 15 (33.33%) with grade 1, 20 (42.22%) with grade 2, 8 (17.77%) with grade 3, and 3 (6.66%) with grade 4. The prevalence of IVH was comparable in female (20; 44.44%) and male (25; 55.56%) neonates with no statistical significance. The risk of IVH grades 1 to 4 was as follows: the greater the lower the gestational age and was significantly higher in children born from 24 + 0 to 28 + 6 weeks of gestation than those born from 29 + 0 to 32 + 0 weeks of gestation (35 (77.78%) vs. 10 (22.22%); *p* = 0.00002); the greater the lower birth weight (*p* = 0.0003); the greater the lower Apgar score in the 1st and 5th minutes of life (*p* = 0.0005); more often in children born vaginally (*p* = 0.023); and more often in children diagnosed with intrauterine infection (*p* = 0.0009). Statistical analysis revealed that IVH grades 3 and 4 occur in lower weeks of gestation, most frequently in children born from 24 + 0 to 28 + 6 weeks of gestation (*p* = 0.022) with lower Apgar scores in the 5th minute of life (*p* = 0.009). Demographic and clinical characteristic of enrolled infants is shown in Table 2.

Our study revealed that the following genotypes were more prevalent in infants who developed IVH: *GC* (OR 1.379; 0.45–4.416) and *CC* (OR 1.625; 0.379–7.013) Il-6 *−174G>C*; *GA* (OR 1.754; 0.587–5.504) and *AA* (OR 1.667; 0.384–7.223) Il-6 *−596G>A*; and *CT* IL-1β *+3953* *C>T* (OR 0.674; 0.253–1.761). Similarly, in infants born from 24 + 0 to 28 + 6 weeks of gestation, the following genotypes had greater prevalence: *GC* (OR 2.6; 0.529–12.93) and *CC* (OR 4.8; 0.294–275) Il-6 *−174G>C*; *GA* (OR 2.7; 0.551–13.39) and *AA* (OR 3.6; 0.191–219.9) Il-6 *−596G>A*; and CT IL-1β *+3953* *C>T* (OR0.4; 0.099–1.625). These findings, however, were not statistically significant. Analysis showed higher prevalence of grade 3 and 4 IVH in children born from 24 + 0 to 32 + 0 weeks of gestation with the following genotypes: *GC* (OR 1.644; 0.298–16.9) Il-6 *+3953C>T*; *GA* (OR 1.853; 0.338–18.93) Il-6 *−596G>A*; and *2/2* Il-1RN (OR 1.917; 0.154–15.15). This same correlation was not present in children born from 24 + 0 to 28 + 6 weeks of gestation. Our investigation also revealed that infants with genotype *1/2* Il-1RN may have higher prevalence of grade 3 and 4 IVH (born from 24 + 0 to 32 + 0 weeks of gestation (OR 2.054; 0.401–11.17) and born from 24 + 0 to 28 + 6 weeks of gestation (OR 3.636; 0.568–26.85)). These results were not statistically significant. Genotype distribution of the polymorphisms in infants without and with IVH or without/with IVH grades 1–2 and with IVH grades 3–4 is presented in Table 3 as well as in infants born between 24 + 0 and 28 + 6 weeks of gestation without and with IVH or without/with IVH grades 1–2 and with IVH grades 3–4 are presented in Table 4. The analysis did not show any higher prevalence of studied genotypes in infected and non-infected patients with IVH (Table 5).

**Table 3:** Genotype distribution of the five polymorphisms in infants without and with IVH or without/with IVH grades 1–2 and with IVH grades 3–4

Gene symbol	Group without IVH *N* = 55 (%)	Group with IVH grades 1–4 *N* = 45 (%)	*p* value	OR	Group without IVH and IVH grade 1 and 2 *N* = 89 (%)	Group with IVH grades 3–4 *N* = 11 (%)	*p* value	OR
IL-1β *+3953* *C>T*(rs1143634)								
Genotype								
*CC* obs exp	32 (58.18) 31.31	30 (66.67) 28.8	–	References	53 (59.55) 51.19	9 (81.82) 9.06	–	References
*CT* obs exp	19 (34.55) 20.37	12 (26.67) 14.4	0.509	0.674 (0.253–1.761)	29 (32.58) 32.61	2 (18.18) 1.82	0.436	0.406 (0.040–2.173)
*TT* obs exp	4 (7.27) 3.31	3 (6.66) 1.8	1.000	0.800 (0.108–5.178)	7 (7.87) 5.19	0 (0.00) 0.09	0.716	0.000 (0.000–4.916)
H-W	0.883	0.535		H-W	0.579		0.946	
Allele								
*C*	83 (75.45)	72 (80)	–	References	135 (75.84)	20 (90.9)	–	References
*T*	27 (24.55)	18 (20)	0.553	0.769 (0.367–1.586)	43 (24.15)	2 (9.1)	0.596	0.628 (0.148–2.024)
Genotype								
*1/1* obs exp	28 (50.91) 25.83	22 (48.89) 19.78	–	References	46 (51.69) 41.86	4 (36.36) 3.84	–	References
IL-1RN *86 bp VNTR* (rs2234663)								
*1/2* obs exp	18 (32.73) 22.34	15 (33.33) 19.44	1.000	1.061 (0.398–2.807)	28 (31.46) 36.28	5 (45.45) 5.32	0.501	2.054 (0.401–11.17)
*1/3*	1 (1.82)	1 (2.22)	1.000	1.273 (0.016–103.4)	2 (2.25)	0 (0.00)	1.000	0.000 (0.000–71.11)
*2/2* obs exp	7 (12.73) 4.83	7 (15.51) 4.78	0.920	1.273 (0.325–4.957)	12 (13.48) 7.86	2 (18.18) 1.84	0.785	1.917 (0.154–15.15)
*2/3*	1 (1.82)	0 (0.00)	1.000	0.00 (0.00–51.41)	1 (1.12)	0 (0.00)	1.000	0.000 (0.000–458.3)
H-W	0.368	0.317		H-W	0.106	0.981		
Allele								
*1*	75 (68.18)	60 (66.66)	–	References	122 (68.53)	13 (59.1)	–	References
*2*	33 (30)	29 (32.22)	0.879	1.098 (0.573–2.097)	53 (29.77)	9 (40.9)	0.437	1.594 (0.563–4.308)
*3*	2 (1.81)	1 (1.11)	1.000	0.625 (0.010–12.31)	3 (1.68)	0	1.000	0.00 (0.000–24.38)
IL-6 *-174* *G>C*(rs1800795)								
Genotype								
*GG* obs exp	13 (23.64) 16.32	8 (17.78) 8.60	–	References	19 (21.35) 22.75	2 (18.18) 3.84	–	References
*GC* obs exp	33 (60.00) 28.36	28 (62.22) 20.81	0.718	1.379 (0.450–4.416)	52 (58.43) 44.49	9 (81.82) 5.32	0.850	1.644 (0.298–16.9)
*CC* obs exp	9 (16.36) 12.32	9 (20.00) 12.60	0.672	1.625 (0.379–7.013)	18 (20.22) 21.75	0 (0.00) 1.84	0.567	0.000 (0.000–6.185)
H-W	0.209	0.081		H–W	0.282	0.072		
Allele								
*G*	59 (53.63)	44 (48.88)	–	References	90 (50.56)	13 (59.1)	–	References
*C*	51 (46.36)	46 (51.11)	0.599	1.209 (0.667–2.195)	88 (49.43)	9 (40.9)	0.599	0.708 (0.254–1.899)
IL-6 *−596* *G>A*(rs1800797)								
Genotype								
*GG* obs exp	15 (27.28) 16.91	8 (17.78) 11.25	–	References	21 (23.60) 24.29	2 (18.18) 3.84	–	References
*GA* obs exp	31 (56.36) 27.17	29 (64.44) 22.50	0.388	1.754 (0.587–5.504)	51 (57.30) 44.41	9 (81.82) 5.32	0.721	1.853 (0.338–18.93)
*AA* obs exp	9 (16.36) 10.91	8 (17.78) 11.25	0.647	1.667 (0.384–7.223)	17 (19.10) 20.29	0 (0.00) 1.84	0.649	0.000 (0.000–7.201)
H-W	0.579	0.153		H–W	0.375	0.072		
Allele								
*G*	61 (55.45)	45 (50)	–	References	93 (52.24)	13 (59.1)	–	References
*A*	49 (44.55)	45 (50)	0.531	1.245 (0.686–2.261)	85 (47.75)	9 (40.9)	0.707	0.758 (0.271–2.031)
TNF-α﻿*−308﻿G>A﻿﻿* (rs1800629)								
Genotype								
*GG* obs exp	42 (76.36) 42.77	35 (77.78) 35.56	–	References	69 (77.53) 70.12	8 (72.73) 8.20	–	References
*GA* obs exp	13 (23.64) 11.46	10 (22.22) 8.89	1.000	0.923 (0.320–2.603)	20 (22.47) 17.75	3 (27.27) 2.59	0.973	1.294 (0.202–60.51)
*AA* obs exp	0 (0.00) 0.77	0 (0.00) 0.56	–	–	0 (0.00) 1.12	0 (0.00) 0.20	–	–
H-W	0.610	0.704			0.490	0.872		
Allele								
*G*	97 (88.18)	80 (88.88)	–	References	158 (88.76)	19 (86.36)	–	References
*A*	13 (11.82)	10 (11.11)	1.000	0.933 (0.346–2.443)	20 (11.23)	3 (13.63)	0.957	1.247 (0.217–4.823)

*Obs* observed, *Exp* expected (genotype frequencies calculated from allele frequencies with the Hardy–Weinberg (H-W) equation)

**Table 4:** Genotype distribution of polymorphisms in infants born 24 + 0–28 + 6 weeks of gestation without and with IVH or without/with IVH grades 1–2 and with IVH grades 3–4

Gene symbol	Group without IVH *N* = 19 (%)	Exp	Group with IVH grades 1–4 *N* = 35 (%)	Exp	*p* value	OR	Group without IVH and IVH grade 1 and 2 *N* = 44 (%)	Exp	Group with IVH grades 3–4 *N* = 10 (%)	Exp	*p* value	OR
IL-1β *+3953* *C>T*(rs1143634)												
Genotypes												
*CC*	10 (52.63)	10.32	25 (71.43)	24.03	–	References	27 (61.36)	26.27	8 (80.00)	8.10	–	References
*CT*	8 (42.11)	7.37	8 (22.86)	9.94	0.243	0.4 (0.099–1.625)	14 (31.82)	15.45	2 (20.00)	1.80	0.648	0.482 (0.045–2.939)
*TT*	1 (5.26)	1.32	2 (5.71)	1.03	1.000	0.8 (0.038–51.86)	3 (6.82)	2.27	0 (0.00)	0.10	0.963	0 (0.000–9.542)
H-W		0.933		0.513				0.823		0.940		
Allele												
*C*	28 (73.68%)		58 (82.85%)		–	References	68 (77.27%)		18 (90%)		–	References
*T*	10 (26.31%)		12 (17.15%)		0.377	0.579 (0.202–1.703)	20 (22.72%)		2 (10%)		0.442	0.425 (0.044–2.078)
IL-1RN*86 bp VNTR* (rs2234663)												
Genotypes												
*1/1*	9 (47.37)	6.72	18 (51.43)	16.46	–	References	24 (54.55)	20.24	3 (30.00)	3.03	–	References
*1/2*	4 (21.05)	8.56	12 (34.29)	15.09	0.827	1.5 (0.318–8.165)	11 (25.00)	18.52	5 (50.00)	4.95	0.219	3.636 (0.568–26.85)
*1/3*	0 (0.00)		0 (0.00)		–	–	0 (0.00)		0 (0.00)		–	–
*2/2*	5 (26.32)	2.72	5 (14.29)	3.46	0.579	0.5 (0.089–2.852)	8 (18.18)	4.24	2 (20.00)	2.03	0.824	2 (0.140–20.59)
*2/3*	1 (5.26)		0 (0.00)		0.714	0 (0.000–21.67)	1 (2.27)		0 (0.00)		1.000	0 (0.000–325)
H-W		0.078		0.481				0.029		0.999		
Allele												
*1*	22 (57.89%)		48 (61.53%)		–	References	59 (67.04%)		11 (55%)		–	References
*2*	15 (39.47%)		22 (38.46%)		0.464	0.672 (0.272–1.683)	28 (31.81%)		9 (45%)		0.368	0.383 (0.039–1.952)
*3*	1 (2.63%)		0		0.648	0.000 (0.000–18.69)	1 (1.13%)		0		1.000	0.000 (0.000–212.7)
IL-6﻿*−174﻿﻿G>C﻿* (rs1800795)												
Genotypes												
*GG*	6 (31.58)	7.58	5 (14.29)	9.26	–	References	9 (20.45)	13.09	2 (20.00)	3.60	–	References
*GC*	12 (63.16)	8.84	26 (74.29)	17.49	0.300	2.6 (0.529–12.93)	30 (68.19)	21.82	8 (80.00)	4.80	1.000	1.2 (0.186–13.55)
*CC*	1 (5.26)	2.58	4 (11.43)	8.26	0.462	4.8 (0.294–275)	5 (11.36)	9.09	0 (0.00)	1.60	0.917	0 (0.000–12.15)
H-W		0.298		0.016				0.045		0.108		
Allele												
*G*	24 (63.15%)		36 (51.42%)		–	References	48 (54.54%)		12 (60%)		–	References
*C*	14 (36.84%)		34 (48.57%)		0.333	1.619 (0.672–3.969)	40 (45.45%)		8 (40%)		0.808	0.764 (0.229–2.401)
IL-6﻿*−596﻿G>A﻿﻿* (rs1800797)												
Genotypes												
*GG*	6 (31.58)	7.58	5 (14.29)	9.78	–	References	9 (20.45)	13.64	2 (20.00)	3.60	–	References
*GA*	12 (63.16)	8.84	27 (77.14)	17.44	0.274	2.7 (0.551–13.39)	31 (70.45)	21.72	8 (80.00)	4.80	1.000	1.161 (0.180–13.11)
*AA*	1 (5.26)	2.58	3 (8.57)	7.78	0.677	3.6 (0.191–219.9)	4 (9.10)	8.64	0 (0.00)	1.60	1.000	0 (0.000–15.59)
H-W		0.298		0.005				0.018		0.108		
Allele												
*G*	24 (63.15%)		37 (52.85%)		–	References	49 (55.68%)		12 (60%)		–	References
*A*	14 (36.84%)		33 (47.14%)		0.408	1.529 (0.634–3.750)	39 (44.31%)		8 (40%)		0.838	0.779 (0.234–2.448)
TNF-α ﻿*−308﻿G>A﻿﻿*(rs1800629)												
Genotypes												
*GG*	16 (84.21)	16.12	28 (80.00)	28.35	–	References	36 (81.82)	36.36	8 (80.00)	8.10	–	References
*GA*	3 (15.79)	2.76	7 (20.00)	6.30	1.000	1.333 (0.256–9.06)	8 (18.18)	7.27	2 (20.00)	1.80	1.000	1.125 (0.098–7.372)
*AA*	0 (0.00)	0.12	0 (0.00)	0.35	–	–	0 (0.00)	0.36	0 (0.00)	0.10	–	–
H-W		0.933		0.806				0.803		0.940		
Allele												
*G*	35 (92.1%)		63 (90%)		–	References	80 (90.9%)		18 (90%)		–	References
*A*	3 (2.63%)		7 (10%)		1.000	1.296 (0.273–8.236)	8 (9.1%)		2 (10%)		1.000	1.25 (0.118–7.120)

*N* observed, *Exp* expected (genotype frequencies calculated from allele frequencies with the Hardy–Weinberg (H-W) equation)

**Table 5:** Genotype distribution of the polymorphisms in infected and non-infected infants with IVH grades 1–4 or with IVH grades 3–4

Gene symbol	Group with IVH 1–4 infected (*N* = 33; %)	Exp	Group with IVH 1–4 non-infected (*N* = 14; %)	Exp	*p* value	OR	Group with IVH 3–4 infected (*N* = 9; %)	Exp	Group with IVH 3–4 non-infected (*N* = 2; %)	Exp	*p* value	OR
IL-1β +3953C>T (rs1143634)												
Genotypes												
* CC*	22 (66.67)	21.28	8 (66.67)	7.52	–	References	7 (77.78)	7.11	2 (100.0)	2	–	References
*CT*	9 (27.27)	10.44	3 (25.00)	3.96	1.000	1.091 (0.196–7.820)	2 (22.22)	1.78	0 (0.00)	0	–	–
*TT*	2 (6.06)	1.28	1 (8.33)	0.52	1.000	0.727 (0.034–48.21)	0 (0.00)	0.11	0 (0.00)	0	–	–
H-W		0.731		0.703			H–W	0.932		–		
Allele												
*C*	53 (80.30)		19 (79.17)		–	References	16 (88.89)		6 (75.00)		–	References
*T*	13 (19.70)		5 (20.83)		1.000	0.932 (0.265–3.797)	2 (11.11)		2 (25.00)		0.717	0.375 (0.023–6.531)
Genotypes												
*1/1*	15 (45.45)	12.73	7 (58.33)	7.36	–	References	3 (33.33)	2.78	1 (50.00)	1.13	–	References
IL -1RN *86 bp VNTR* (rs2234663)												
*1/2*	11 (33.33)	15.53	4 (33.33)	3.27	1.000	1.283 (0.246–7.497)	4 (44.44)	4.44	1 (50.00)	0.75	1.000	1.333 (0.013–130.5)
*1/3*	0 (0.00)		1 (8.33)		0.696	0.000 (0.000–20.80)	0 (0.00)		0 (0.00)		–	–
*2/2*	7 (21.21)	4.73	0 (0.00)	0.36	–	–	2 (22.22)	1.78	0 (0.00)	0.13	–	–
IL-1RN (rs2234663) *2/3*	0 (0.00)		0 (0.00)		–	–	0 (0.00)		0 (0.00)		–	–
H-W		0.246		0.762				0.956		0.895		
Allele												
*1*	41 (61.19)		19 (82.61)		–	References	10 (55.56)		3 (75.00)		–	References
*2*	25 (38.81)		4 (17.39)		0.115	2.896 (0.819–12.92)	8 (44.44)		1 (25.00)		0.899	2.4 (0.150–141.5)
*3*	0 (0.00)		0 (0.00)		–	–	0 (0.00)		0 (0.00)		–	–
IL-6−*174 G>C* (rs1800795)												
Genotypes												
*GG*	5 (15.15)	7.28	3 (25.00)	3.52	–	References	1 (11.11)	2.78	1 (50.00)	1.13	–	References
*GC*	21 (63.64)	16.44	7 (58.33)	5.96	0.777	1.800 (0.218–12.22)	8 (88.89)	4.44	1 (50.00)	0.75	0.691	8 (0.051–704)
*CC*	7 (21.21)	9.28	2 (16.67)	2.52	0.873	2.1 (0.162–32.92)	0 (0.00)	1.78	0 (0.00)	0.13	–	–
H-W		0.281		0.832			H–W	0.056		0.895		
Allele												
*G*	31 (46.97)		13 (54.17)		–	References	10 (55.56)		3 (75.00)		–	References
*C*	35 (53.03)		11 (45.83)		0.715	1.334 (0.472–3.810)	8 (44.44)		1 (25.00)		0.899	2.4 (0.150–141.5)
IL-6−*596 G>A* (rs1800797)												
Genotypes												
*GG*	5 (15.15)	7.28	3 (25.00)	4.08	–	References	1 (11.11)	2.78	1 (50.00)	1.13	–	References
*GA*	21 (63.64)	16.44	8 (66.67)	5.83	0.888	1.575 (0.196–10.42)	8 (88.89)	4.44	1 (50.00)	0.75	0.691	8 (0.051–704)
*AA*	7 (21.21)	9.28	1 (8.33)	2.08	0.569	4.2 (0.227–251.3)	0 (0.00)	1.78	0 (0.00)	0.13	–	–
H-W		0.281		0.437				0.056		0.895		
Allele												
*G*	31 (46.97)		14 (58.33)		–	References	10 (55.56)		3 (75.00)		–	References
*A*	35 (53.03)		10 (41.67)		0.475	1.581 (0.557–4.583)	8 (44.44)		1 (25.00)		0.899	2.4 (0.150–141.5)
TNF-α*−308G>A* (rs1800629)												
Genotypes												
*GG*	26 (78.79)	26.37	9 (75.00)	9.19	–	References	7 (77.78)	7.11	1 (50.00)	1.13	–	References
*GA*	7 (21.21)	6.26	3 (25.00)	2.63	1.000	0.808 (0.142–5.898)	2 (22.22)	1.78	1 (50.00)	0.75	0.982	0.286 (0.003–33.55)
*AA*	0 (0.00)	0.37	0 (0.00)	0.19	–	–	0 (0.00)	0.11	0 (0.00)	0.13	–	–
H-W		0.793		0.885				0.932		0.895		
Allele												
*G*	59 (89.39)		21 (87.50)		–	References	16 (88.89)		3 (75.00)		–	References
*A*	7 (10.61)		3 (12.50)		1.000	0.831 (0.169–5.441)	2 (11.11)		1 (25.00)		0.940	0.375 (0.015–29.43)

*N* observed, *Exp* expected (genotype frequencies calculated from allele frequencies with the Hardy–Weinberg (H-W) equation)

## Discussion

In our study, we evaluated the possible association between Il-1, Il-6, TNFα, and Il-1RN VNTR gene polymorphisms and the development of IVH, in a large study population of preterm infants born from 24 + 0 to 32 + 0 weeks of gestation with the exposition on AST. AST is administered to women in the case of suspicion of preterm delivery and has been proven to reduce the prevalence and severity of IVH [7–9]. IVH has been attributed to alteration in cerebral blood flow to the germinal matrix, which, as suggested by risk factor studies, might be responsible for the genes contributing to the infection and inflammation response. Inflammatory factors including cytokines such as Il-1, Il-6, and TNFα have been implicated in IVH development [10]. In the population of Polish women, maternal carriage of polymorphic alleles of IL-1β, IL-6 promoter, TNF-α promoter, and IL-1RN seems to have no impact on the risk of preterm delivery due to preterm premature rupture of membranes [11].

Il-1 is produced by activated macrophages and stimulates the production of other pro-inflammatory cytokines, such as interferon-γ, Il-6, and TNFα. Subtype Il-1β is mostly responsible for the pro-inflammatory effects and for hypothalamic–pituitary–adrenal axis activation. Il-1β is developmentally and regionally regulated in the brain of developing fetuses and neonates [12, 13]. Increased levels of Il-1β were observed in amniotic fluid and/or cord blood of infants with perinatal brain injury including IVH and periventricular leukomalacia (PVL) [14]. A previous study by Ryckman et al. demonstrated that infants born before 32 weeks of gestation with the *CT* genotype or the Il-1β *−31* *C* allele were at higher risk for IVH grades 1 and 2 and those with the *CC* genotype or *C* allele were at higher risk for IVH grades 3 and 4 [15]. The Il-1β *−31* allele *C* is associated with increased production of Il-1β in vivo. Baier et al. evaluated the role of Il-1β *511C>T* polymorphism in a population of very low birth weight infants. Compared to 14% with the *C* allele, one third of newborns with the *T* allele developed IVH. The Il-1β *−511T* allele was strongly associated with the IVH grade 3 and 4 development [16]. Aden et al. analyzed Il-1β −*511C>T* polymorphism and Il-1β *−31C*  polymorphism in 224 inborn preterm infants treated with AST and grade 3 and 4 IVH, finding a lack of association with IVH [17]. We investigated the role of Il-1β +*3953C>T* polymorphism in the pathogenesis of IVH. A single-nucleotide polymorphism at position *+3953* of the gene encoding IL-1β, which consists of the replacement of cytosine by thymine, leads to the emergence of a less frequent allele *2*. This allele *2* is associated with increased production of IL-1β [18]. Our data demonstrates that the polymorphism Il-1β *+3953C>T* is not a predictor of an increased risk of IVH grade in preterm infants, which is a new finding in this group of patients.

Il-6 has a wide, bidirectional (pro-inflammatory and anti-inflammatory by acting against Il-1 and TNFα) spectrum of action including nervous system development [19]. The role of Il-6 in preterm brain injury, however, is unclear. No correlation was found between Il-6 concentration in cord blood and IVH development [20–22]. Comparatively, Heep et al. investigated postnatal levels of Il-6 in serum and found that IVH was present significantly more frequently in the group with Il-6 levels above 100 pg/ml [19]. The replacement of guanine by cytosine at position −174 of the IL-6 gene results in diminished production of IL-6 in homozygous *CC* individuals. Similarly, the replacement of guanine by adenosine at position −596 causes decreased IL-6 production by *AA* homozygotes [23]. Hording et al. reported that in preterm neonates, the *CC* genotype of Il-6 −*174* *G>C* polymorphism significantly increased the risk for IVH. These findings, however, have not been confirmed by Gőpel et al., Baier et al., Ryckman et al., and Aden et al. [15–17, 24]. We did not find the association between genotypes of Il-6 −*174* *G>C* polymorphism and IVH. We were not able to confirm any association between genotypes of Il-6 −*596* *G>A* polymorphism either, which itself has not been previously reported.

TNFα is produced by activated macrophages and other cells. TNFα may participate in preterm brain injury in several ways, damaging the microcirculatory endothelium of the central nervous system and therefore increasing the risk of vessel rupture, stimulating the production of tissue factor and coagulation cascade activation, and stimulating the production of platelet-activating factor which can damage cell membranes [25]. The replacement of guanine by adenine at position −308 of the TNF gene promoter region causes the loss of the binding site for the AP-2 transcription factor [26]. The role of polymorphism −308 TNFα is ambiguous. Adcock et al. reported that allele A in the −308 TNFα promoter region is associated with IVH in preterm infants. Similar conclusions were reported in the studies of Heep et al., Ryckamn et al., and Aden et al. [15, 17, 19]. Our analysis did not reveal the positive correlation between TNF * −308G>A* polymorphism with the prevalence of IVH.

The Il-1 receptor antagonist is a protein binding with receptors for Il-1 (Il-1 R1 and Il-1 R2). The VNTR polymorphism has been reported within intron 2 of the human Il-1 receptor antagonist gene, consisting of repeats of 86 bp sequence. The number of repeats is of functional significance as these repeats contain binding sites for transcription factors. The *Il-1 RN1* allele corresponded to a 410 bp fragment (four copies of the −86 repeat), *Il-1 RN0* to 154 bp, *Il-1 RN2* to 240 bp, *Il-1 RN3* to 500 bp, *Il-1 RN4* to 325 bp, and *Il-1 RN5* to 585 bp [27]. It is notable to mention that various alleles are associated with the production of this protein at different levels. *Il-1 RN2* occurrence leads to a prolonged and more intense pro-inflammatory response compared with those who do not carry this genotype [28]. In the present study, we examined the association of VNTR polymorphism of Il-1 RN with the IVH prevalence in preterm infants. To our knowledge, there has been no study investigating Il-1 RN gene polymorphism in preterm infants with IVH thus far. We have not found a correlation between the occurrence of the allele and prevalence of IVH.

The limitations of our study include the relatively small sample size, which could affect the demonstration of correlation between studied complications and polymorphisms. However, given the homogeneity of our population, it is unlikely that the observed associations are a statistical artifact resulting from a population substructure.

## Conclusions

IVH is a significant problem for preterm infants. In addition to little progress in preventing IVH in preterm babies, substantial research that are focused on understanding the etiology, mechanism, and risk factors for IVH are imperative. In the era of personalized medicine, identification of genetic risk factors creates opportunities to generate preventative strategies. Further studies should be performed to confirm the role of genetic factors in etiology and pathogenesis of IVH.

### Contributions

D.S. designed the research and wrote the manuscript. D.S., J.G., A.S-M., G.K., KD., and M.S. performed the research. D.S. collected and analyzed the data, and G.K. was responsible for the PCR procedure.

## Electronic supplementary material

ESM 1 (XLS 27 kb)
